# Integrating an openEHR-based personalized virtual model for the ageing population within HBase

**DOI:** 10.1186/s12911-019-0745-8

**Published:** 2019-01-28

**Authors:** Spyridon Kalogiannis, Konstantinos Deltouzos, Evangelia I. Zacharaki, Andreas Vasilakis, Konstantinos Moustakas, John Ellul, Vasileios Megalooikonomou

**Affiliations:** 1 Computer Engineering and Informatics Department, University of Patras, University Campus, Rio, 26504 Greece; 2 Information Technologies Institute, Centre for Research and Technology Hellas, 6th km Charilaou-Thermi Rd, Thessaloniki, 57001 Greece; 3 Department of Neurology, School of Medicine, University of Patras, University Campus, Rio, 26504 Greece

**Keywords:** Electronic health records, Semantic interoperability, Virtual models, openEHR archetypes, NoSQL database, Frailty

## Abstract

**Background:**

Frailty is a common clinical syndrome in ageing population that carries an increased risk for adverse health outcomes including falls, hospitalization, disability, and mortality. As these outcomes affect the health and social care planning, during the last years there is a tendency of investing in monitoring and preventing strategies. Although a number of electronic health record (EHR) systems have been developed, including personalized virtual patient models, there are limited ageing population oriented systems.

**Methods:**

We exploit the openEHR framework for the representation of frailty in ageing population in order to attain semantic interoperability, and we present the methodology for adoption or development of archetypes. We also propose a framework for a one-to-one mapping between openEHR archetypes and a column-family NoSQL database (HBase) aiming at the integration of existing and newly developed archetypes into it.

**Results:**

The requirement analysis of our study resulted in the definition of 22 coherent and clinically meaningful parameters for the description of frailty in older adults. The implemented openEHR methodology led to the direct use of 22 archetypes, the modification and reuse of two archetypes, and the development of 28 new archetypes. Additionally, the mapping procedure led to two different HBase tables for the storage of the data.

**Conclusions:**

In this work, an openEHR-based virtual patient model has been designed and integrated into an HBase storage system, exploiting the advantages of the underlying technologies. This framework can serve as a base for the development of a decision support system using the openEHR’s Guideline Definition Language in the future.

## Background

During the last decade there has been great interest in systems used for health monitoring and safety [1–5]. One of the key aspects in such systems is to store medical data efficiently in order to capture health status across time. Towards this direction, a number of electronic health records (EHR), or electronic medical records (EMR), have been developed. Some works extend the idea of EHR and focus on developing a personalized virtual patient model (VPM) composed of person’s medical records [6–8].

A factor of high importance for components or systems which require to use data and information exchanged between them [9–11] is the interoperability [10]. Towards this direction, the European Commission’s eHealth Action Plan 2012-2020 promotes semantic interoperability of EHR systems as a crucial challenge in eHealth solutions [12–16] in an effort to provide a roadmap to empower patients and healthcare workers.

Formal modeling of clinical content that can be made available internationally is one of the most promising pathways to semantic interoperability of health information [17]. Over the last two decades many attempts have been made to solve the major issues of health data systems that include semantic interoperability across systems as well as between components of a system, and decision support based on intelligent data analysis. Moreover, computational challenges in health systems due to which standard Information and Communication Technology (ICT) systems are hard to keep up, include the high variety and complexity of data, and high rate of data change ranging from clinical processes to protocols.

In this section we first provide a few details on the Electronic Health Record standardization, followed by a brief overview of DBMS solutions for storing standardized EHRs. Then we focus on the monitoring systems for older adults and how these can be used for assessing frailty.

### Electronic health record standardization

An EHR is an electronic version of a patient’s medical history, that is maintained by a healthcare provider over time. It usually includes all the key administrative clinical data relevant to that persons’ care under the particular provider, including demographics, progress notes, problems, medications, vital signs, past medical history, immunizations, laboratory data and radiology reports [18].

Different international organizations have worked on the definition of an EHR architecture. Health Level 7 (HL7) [19] is a set of international standards for clinical data that focus on the application layer, which is "layer 7" in the OSI model. These standards are produced by an international standards organization (HL7 International), and are widely adopted by other bodies such as American National Standards Institute and International Organization for Standardization. The HL7 standards are basically a set of rules that allow clinical information to be shared and processed in a uniform and consistent manner.

The Health Informatics Technical Committee (TC251) of the European Committee for Standardization (CEN/ TC251) [20] has completed a European Standard for the communication of the EHR, called CEN EN13606 whose reference model became an ISO standard in February 2008 under the name ISO 13606. Exploiting this ISO, the openEHR consortium [21] maintains an architecture designed to support the constructions of distributed, patient-centered, life-long, shared care health records. OpenEHR is an open standard specification that is used to facilitate storage, management, retrieval and exchange of health data between different healthcare providers or other interest groups. In openEHR, all health data for a person are stored in a lifelong, person-centered EHR. The openEHR specifications include an EHR Extract specification [22] but in contrast to other standards such us EN 13606 and HL7, the exchange of data between EHR-systems is not their main primary concern. The openEHR framework will be discussed in detail in the “Methods” section.

Since the purpose of this work is to exploit openEHR archetypes and to adapt the openEHR framework for the representation of frailly in older person models, similar works which investigate or adapt the openEHR model for the representation of the required entities are presented next. In one of the earliest works [23], the modeling of a prototype neonatology Electronic Patient Record using openEHR archetypes was introduced, while more recently [24] the suitability of openEHR with its reference model, archetypes and templates, for the digital representation of demographic information and obstetrics related clinical data was investigated. Also the openEHR was elaborated as a tool for modeling Hospital Information Systems on a regional level based on a national logical infrastructure. Other noticeable works include the archetypes reuse for modeling the EHR of children affected by cerebral palsy [25], the adoption of the openEHR standard for the development of an EHR for methadone treatment recording and decision support [26], the validation of openEHR archetypes for the Multiple Sclerosis Functional Composite [27] and the modeling of healthcare authorization and claim submissions using the openEHR dual-model approach [28]. Even though openEHR has been exploited by several researchers and developers, none of the existing solutions has investigated its use for the comprehensive formal modeling of older people’s health, making this an important research issue.

### DBMS solutions for storing standardized EHRs

It is generally agreed that the storage and management of medical data is a task of high difficulty, as clinical data are dynamic, sporadic, heterogeneous and complex in nature [29, 30]. As mentioned in [31], despite the fact that medical data share some of the characteristics of the typical data managed by conventional Database Management Systems (DBMS), special attention is required in the design of the corresponding database schemas, due to the unique features that the medical data possess. The selection of database and its corresponding schema design are factors that affect the effective management of clinical data (query performance, scalability, flexibility and extensibility) which usually emerges, especially in systems developed for real-time usage.

The most widely used approach in object-oriented systems is the Object-Relational Mapping (ORM) [32] which is essentially a relational model that allows users to integrate object-oriented features into it. Regarding its application in databases used for storing standarized EHRs, it aims at an exhaustive mapping between the structure of the EHR and the relational database. However, the complex nature and the variability of the information represented by the openEHR model makes the application of ORM in archetype-based systems a complicated and inefficient option, as complicated queries are required even for the retrieval of simple information, leading to low performance [33–35].

A remarkable persistence solution, also based on the relational paradigm, has been proposed by openEHR. This method is called "Node and Path" [36] and uses the relational model to store BLOBs (binary large objects that store serialized subtrees of the archetype XML file extract) into relational tables based on semantic paths defined by the archetypes structure. This method is adopted by numerous archetype-based systems, as mentioned in a survey [37] concerning openEHR storage implementations worldwide until 2013. The wide adoption of a relational persistence solution may be a result of the domination of the relational paradigm in the DBMS field due to its simplicity and hence, the developers’ long experience in it. Nevertheless, its simplicity results in complex queries, which convert data retrieval into an inefficient task [34, 36].

Another notable solution proposed by Wang et al. [38] is Archetype Relational Mapping (ARM), which builds a relational database schema driven by mappings between the openEHR archetypes and relational tables, in contrast with Node + Path which aims at an archetype-independent data storage structure. The comparison conducted in this work between archetype-relational mapping and the BLOB approach showed that the performance of the two methods was similar for single-patient queries, but the performance of ARM was much more efficient for population-based queries.

In other archetype-based implementations, as also mentioned by Frade et al. [37], the adopted storage method was an XML database, which provides flexibility and compatibility advantages. However, XML databases are not capable of providing satisfactory response time when population-based queries are submitted to large datasets, in comparison to their efficiency in single-patient queries [39].

Investigating solutions to overcome the drawbacks coming of the aforementioned relational and XML persistence methods, the NoSQL approach has been examined or adopted in few openEHR-based implementations [31, 33, 34, 40]. NoSQL databases provide a mechanism for storage and retrieval of data which are modeled by means other than the tabular relations used in relational databases. Some of their basic characteristics are that they are schema-free, distributed, open-source, low-cost, horizontally scalable and can store a huge amount of data. All the related works reach a common observation: the NoSQL databases outperform the relational and the XML models in terms of characteristics. More specifically, the results in [31] show that a NoSQL database (produced by MS-SQL) is the best choice for query speed compared to an XML database, while in another comprehensive work [33] the disadvantages of XML and relational databases are pointed out, as experiments showed that the implemented NoSQL database (Couchbase) had better response times than the relational database and both were faster than the XML one. Therefore, the NoSQL database seems to be a promising solution for retrieving results from population-based queries in openEHR-based systems. In another work that examines database persistence of ISO/EN 13606 standardized electronic health record extracts, the comparison of relational and NoSQL approaches, also led the researchers into the conclusion that NoSQL databases perform better in concurrency, using the MongoDB NoSQL database [41].

The NoSQL databases (Couchbase, MongoDB) used in all the aforementioned works are document-based, which pair each key with a complex data structure known as a document. These documents can contain many different key-value pairs, or key-array pairs, or even nested documents. On the other hand, in wide-column or column-family databases, which are a different type of NoSQL databases, data are managed differently. The data are stored in cells identified by a rowkey and a column name, and each column belongs to a column family. While column families need to be defined on the creation of the table, the columns can be created dynamically at runtime. This results in a very flexible schema that can host a virtually unlimited number of columns with the advantage of very fast access/search and data aggregation. The most famous databases of this category are Google’s BigTable, as well as HBase and Cassandra that were inspired from BigTable.

### Monitoring systems for elderly

Recently, special attention has been given in electronic technologies that support ageing of older adults in their home environment [42]. There is a significant number of surveys focusing on monitoring systems for older adults, including wearable ECG monitoring systems [3], and tools and technologies in ambient-assisted living [43]. A review on the acceptance of technology for ageing is provided in [44]. The emphasis in monitoring older adults, comes as a result of the constant increase of the ageing population which affects the planning and delivery of health and social care. The age related decline is characterized by reduced physical, physiological and cognitive function, and results to frailty. Frailty is a common clinical syndrome in ageing population that comes as a result of cumulative declines across multiple physiologic systems, and carries an increased risk for adverse health outcomes including falls, hospitalization, disability, and mortality [45]. In order to prevent these adverse outcomes there is the need of monitoring the physiological clinical state of older people using both embedded and behavior monitoring sensors. By integrating such sensor data in an standardized electronic health system, one could achieve efficient interconnection with other heterogeneous systems.

### Proposed framework for monitoring frailty

This paper aims to create a modeling approach for the health of the ageing population, using existing or newly developed openEHR archetypes. This is an innovative application since none of the previous works has attempted to describe the required entities for older people’s frailty status using the openEHR model. Additionally, we describe a methodology for a mapping between openEHR archetypes and a wide-column NoSQL database. Such a mapping has not been presented in depth before, although there are some works [46] which use HBase to represent electronic health records.

Our work is part of the FrailSafe project [47] which aims to develop a safe and unobtrusive real life sensing (physical, cognitive, psychological, social, etc) and intervention platform for the ageing population using advanced data analysis tools [48–50], considering that frailty has major health care implications and all persons older than 70 years should be screened for frailty [45].

## Methods

In our representation framework we selected the openEHR model since it is considered as one of the most representative approaches available for realising the goals of monitoring and decision support systems and the only open specification available which meets all requirements for complete EHR interoperability in contrast to most EHR standards [51].

By utilizing the openEHR modeling approach for FrailSafe, virtual modeling of older people can be performed using a stable reference model (RM) [22] as a general framework, and this direction can be advantageous from multiple points of view:

1. It enables the use of archetypes as domain information to achieve greater flexibility and stability, as they can efficiently be adopted in problems like frailty prediction, in which the domain concepts are vast in number, have complex relationships, and evolve continuously.
2. The openEHR RM ensures that VPM can always send information to other modules and receive readable information in return, thus ensuring data interoperability between different components of a platform or multiple platforms.
3. The summary of archetypes (reused, newly developed and modified) from this work are shared in a public open source repository [52] and can be used by other researchers/clinicians/developers for the representation of clinical information that can be processed by their systems and thus this work enables semantic interoperability [53] for ageing-related systems that will be developed in the future. Since none of the known works has been focused on finding or developing archetypes for modeling the older people’s health, our research can be considered pioneering and advantageous from this point of view.
4. From an implementation point of view, the specialists’ responsibilities from the ICT domain and the healthcare domain are disengaged. Developers focus only on the technical components of VPM system, while healthcare specialists develop the structural model based on domain concepts archetypes. This enables domain specialists to directly organize and present healthcare information and interactions, and to control the framework without intervention from the system supplier.
5. The VPM coupled with several modules and tools (i) facilitates the analysis of the collected data and feature extraction, (ii) supports the physician in his/her decision process ranging from general health preservation monitoring to critical situation management, and (iii) allows a personalized feedback to the older person which ultimately can be linked with lifestyle change suggestions, behavior guidelines and medical intervention strategies.
6. The methodology for the one-to-one mapping between openEHR archetypes and the column-families NoSQL schema described in this paper is shared in a public open source repository [52] and can be considered as a notable guide for others, as not many works have focused on the use of NoSQL solutions in archetype-based systems and more specifically none of them has described a methodology for the aforementioned mapping.

In this section we give more details on the incorporated technology, by providing a detailed overview of the openEHR model. The entities and concepts of interest regarding frailty in ageing population are presented and the main characteristics of the virtual patient model are briefly introduced. The most highly-related existing openEHR archetypes that best fit the identified parameters are explored and entirely new archetypes are designed and created, or existing ones are extended/specialized for the rest of the parameters. Finally the generated archetype-based virtual patient model storage in a NoSQL (Not Only SQL) (SQL, Structured Query Language) database is discussed in detail and the methodology followed for this procedure is analyzed.

### OpenEHR

OpenEHR is based on the "two-level" modeling approach [54]; the first level of modeling is composed by the RM, which is the information reference model that defines the semantics of data; the second level includes formal definitions of clinical knowledge in the form of archetypes and templates. Only the RM is implemented in software, therefore this dual-level methodology enables the separation of the clinical content definitions from the software, by making the developed systems independent of variable content definitions. As a result, openEHR-based systems have the possibility of being smaller than "single-level" systems, as well as self-adapting, since archetypes and templates can be easily integrated to them without significant modifications [55].

Archetypes are the backbone of the openEHR and they can be described as specifications which define formally in a machine readble format how clinical data can be stored. Each archetype is a model for the description of a particular clinical concept. Archetypes can be divided in 4 different classes [56]:

1. COMPOSITION class is the most abstract class in the openEHR model. In practice, it could be used to contain all information stored in an EHR, as it includes high level (abstract) knowledge about other more descriptive clinical entities which can be added under this class. It could be seen as a piece of paper containing only keywords without any specific details.
2. SECTION class is included in a COMPOSITION most of the times, as it is the second most abstract class and it has the same role as sections in any document. More specifically, sections don’t bear detailed information, but they are used to provide the frameworks in which different kinds of Entry and Cluster class archetypes are nested. For example, Vital Signs and Physical Examination correspond to two different Sections.
3. ENTRY class is an autonomous unit of information regarding elements that can be grouped together to describe a particular clinical entity (e.g. blood pressure, heart rate etc.). The meaning of this information remains unchanged in any system or settings it is used. There are 4 subcategories of the ENTRY class:
  Observations: they correspond to raw information, as they have been measured by devices, reported by patients or noticed by doctors (test findings, symptoms noticed etc.).
  Evaluations: they include interpreted clinical information (in contrast to Observations which are uninterpreted) which have been been assessed by doctors. In this subclass, the knowledge knowledge is extracted from the initial measurements leading to a conclusion. For instance, Problem/Diagnosis archetype belongs to this category.
  Instructions: they refer to commands about future that should be performed or steps that should be followed. A typical example of this archetype category is a medication order.
  Actions: they refer to activities that have already taken place. Actions are often used to record in what extent the Instructions have been followed.
4. CLUSTER Class is used for the description of entities which are important for the modeling of many clinical concepts or scenarios and that is why archetypes of this category can be utilized in any ENTRY or other CLUSTER archetypes. Their use is characterized by recursiveness. Common examples are symptom, size, body location etc.

In addition, there is a type of archetypes, based on an Information Model named DEMOGRAPHIC [22] which includes archetypes for the modeling of demographic information.

Archetypes are expressed with the use of a formal language named Archetype Definition Language (ADL) [57]. ADL is designed as a human-readable and computer-processible syntax, used in the same way as a programming language syntax is used to represent programming constructs.

### FrailSafe virtual patient model research methodology

#### Requirements

The requirements necessary to meet the objectives of our study (FrailSafe project [47]) and the basic characteristics of the corresponding virtual patient model representation scheme are presented in detail in this subsection. The requirement analysis was performed by our clinical partners (Department of Neurology - University of Patras [58], Materia Group [59], Inserm [60]) after studying the state-of-the-art of frailty and selecting a set of clinical entities that constitutes a coherent virtual patient model for monitoring health status and preventing age-related decline. The entities were selected following the extended classical clinical evaluation, namely the comprehensive geriatric assessment (CGA) [61] and augmented with measurements from novel high technology instruments, constituting the FrailSafe system. The CGA quantifies frailty by evaluating a person’s medical history and prescription, cognitive and emotional status, physiological, social and other life conditions. The quantification of these aspects of a person’s global health condition is done by using questionnaires, standardized scales and cumulative indices [62–68], and this evaluation is repeated throughout the study’s duration. In order to avoid selection bias, all major variables from each domain (physiological, cognitive, etc) were used to define the clinical entities. In general the VPM is designed to be personalized, in a sense that the frailty related entities are categorized into data related to the user identification, medical data essential to the clinicians, summary of the integrated sensor recordings, and analyzed data.

The entities can be divided into four main classes which are further separated into subcategories:

1. Personal details: This category includes all the requirements which are related to the patient’s personal information. It can be divided into three subcategories:
  Identification details: This subclass involves all the requirements which are related to the identification of the person, such as his/her name.
  Demographics details: This subclass involves all the requirements concerning demographic information, such as gender and age.
  Contact details: This category relates to the documentation of contact details of the patients or older people.
2. Monitoring parameters: This category includes the representation of the collected data from medical questionnaires, sensing devices and serious games used to access the older people’s cognitive and physiological state. More specifically, the data can be distributed in the following subcategories:
  Physiological measurements, such as heart rate or respiratory rate.
  Physical/functional measurements, such as motor and strength condition.
  Psychological measurements, such as depression and anxiety.
  Social interaction and behavioral parameters, such as number of phone calls per week.
  Cognitive measurements, such as progress in VR/AR games and deficiencies in electronic written text.
  Lifestyle parameters, such as alcohol consumption and indoor/outdoor activity levels.
  Nutrition-dependent entities, such as body mass index (BMI) and body fat.
  General condition, such as unintentional weight loss.
  Wellness, such as self-rated health status.
  Environmental factors, such as number of steps to access house.
  Medical domain, such as number of co-morbidities.
  Frailty metric.
3. Events: This category includes a variety of events to be recorded during the monitoring of health-related entities in cases of abnormalities or critical changes. All the produced events should be stored while the most critical ones should trigger an alert towards the clinician, the older people and their closest family members. The produced alerts can be categorized in two different classes:
  Alerts related to detection or prediction of acute or sudden events, such as instability or fall.
  Notifications about change or critical evolution of a clinical metric.
4. Personalized interventions including documentation of care plans, medication, as well as lifestyle and game recommendations offered by the clinicians to the older people, based on their monitored health status and lifestyle.

The acquired data can be classified according to their sampling frequency into two categories: static data that do not change over time, such as patient identification, demographic information and contact details and dynamic data in the form of continuous data streams such as the sensor recordings which might have significant predictive value for frailty, or dynamic data aggregated within a fixed sampling window, such as the data essential for clinical diagnosis and intervention. The determination of the requirements is followed by the definition of coherent and clinically meaningful parameters that cover every aspect of the concepts to be represented.

#### Archetype-based entities representation

This subsection presents the methodology used for modeling FrailSafe’s entities using openEHR archetypes. In an effort to create a personalized modeling framework based on the openEHR platform, the most relevant archetypes need to be retrieved from the openEHR clinical knowledge manager (CKM) in respect to the requirements presented in the Requirements subsection. Through this mapping procedure between FrailSafe’s parameters and existing archetypes, three cases can be distinguished:

1. An existing archetype can be reused if all objects of a parameter have already been modeled in it. Most of the times, the existing archetypes contain more objects than needed, as they aim to a complete representation of the requested concept. In these cases, the unused objects of the archetype can be excluded via templates.
2. If a parameter cannot be represented by an existing archetype due to the lack of particular fields in it, this archetype can be modified by adding the missing objects.
3. If no appropriate archetype exists for modeling the required parameters, a new archetype has to be created using the openEHR Archetype Editor [69].

As described in the openEHR subsection, openEHR archetypes can be divided into particular categories, each of them being used for modeling different parts of the clinical recordings and workflow processes.

**Fig. 1:**
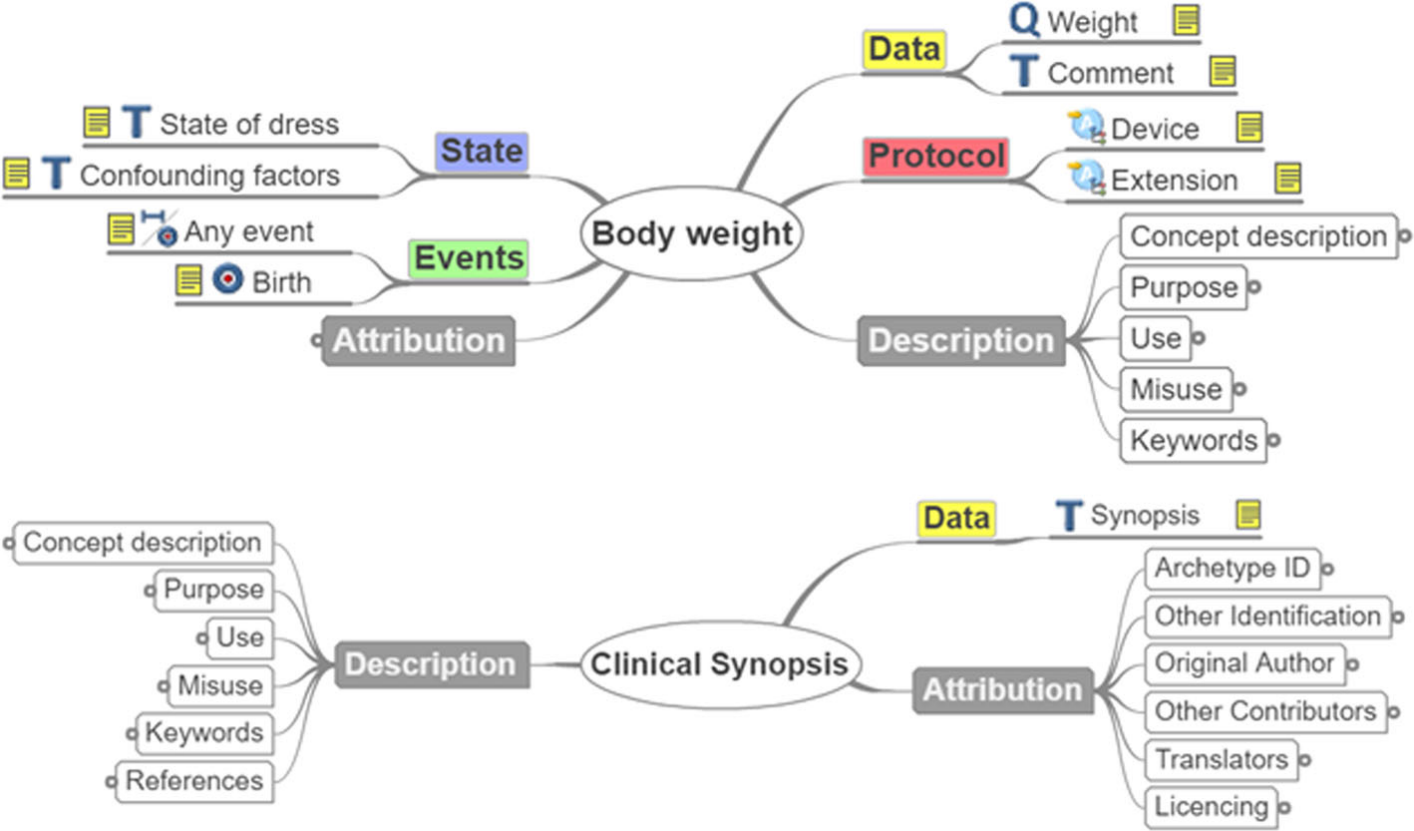
Typical examples of openEHR archetypes structure

Figure 1 illustrates the structure of the archetypes “EHROBSERVATION.body_weight.v2” and “EHR-EVALUATION.clinical_synopsis.v1" which are typical examples of the categories "Observations" and "Evaluations" respectively. These figures are called mindmaps and are generated by the openEHR CKM online tool. The concepts described by each archetype are divided into specific categories based on the archetype class the archetype belongs to. These categories can be considered as labels for the contents of the archetype. For instance, the labels of the objects in the archetype “EHR-OBSERVATION.body_weight.v2” as in every Observation archetype are: State, Events, Data, Protocol, Description, Attribution.

#### Integration of archetypes into a NoSQL database system

In this subsection the methodology for a one-to-one mapping between openEHR archetypes and a column-family NoSQL schema is described.

We selected Apache HBase for the storage of the data of our project, as column-family databases are the most suitable for the nature of our case study. More specifically, in FrailSafe project except for the abstract medical information, sensor raw data is also collected in a real-time scenario. The frequency of the measurements is very high (e.g. 25Hz), hence the DBMS has to be capable of managing hundreds of gigabytes of data efficiently in real-time. HBase is considered as the most suitable option as: (1) it is distributed and scalable, (2) it is optimized for fast read/write access to Big Data, (3) it can host very large tables with billions of rows × millions of columns [70].

In HBase only column families are defined by the table schema. A table can consist of multiple column families, each of which can have any number of columns. In general, the table design logic in HBase is the following: (1) A table is a group of rows, (2) A Row is a group of column families, (3) A column family is a group of columns, (4) A column is a group of key value pairs [71].

**Fig. 2:**
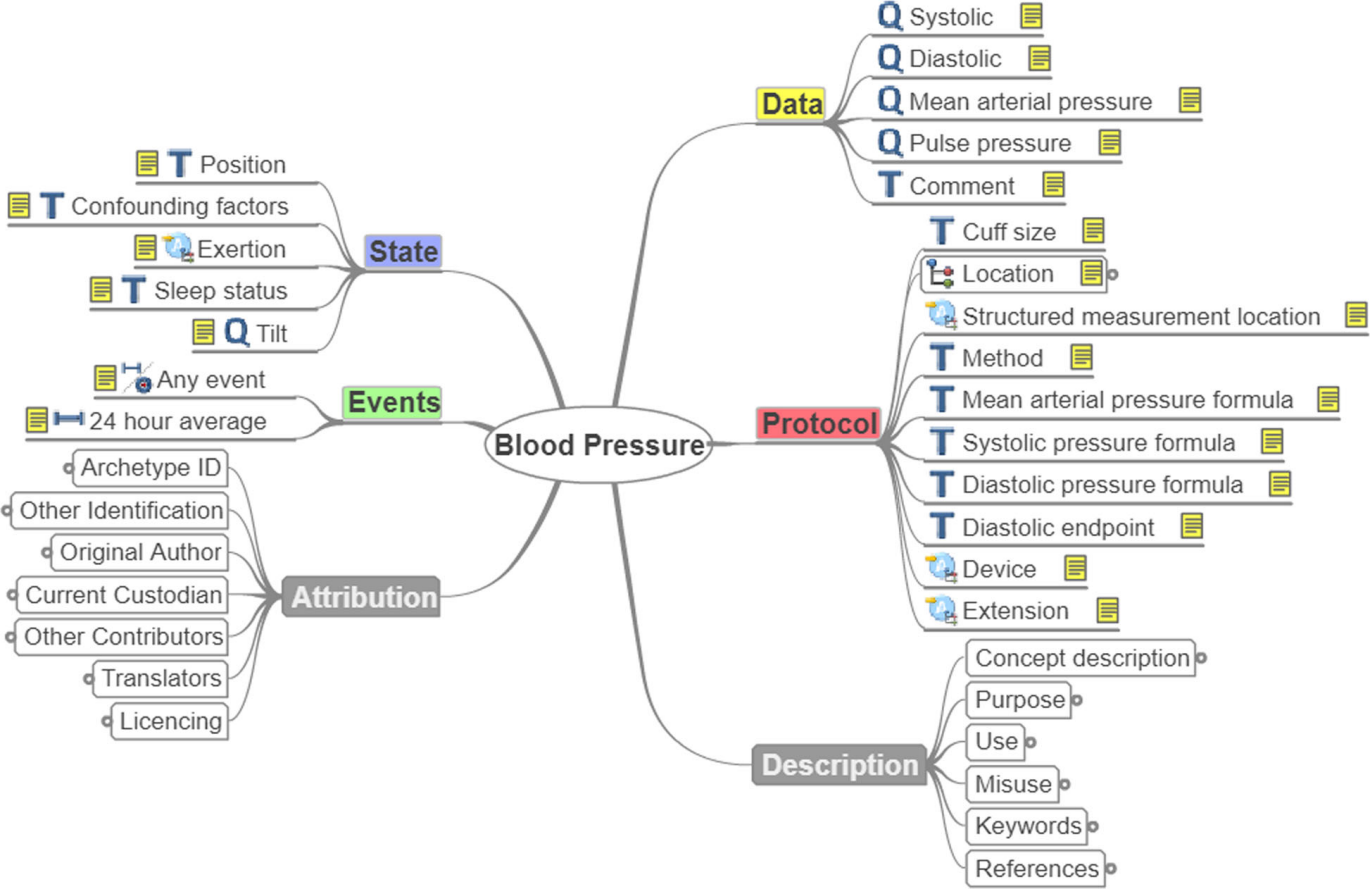
Example of reused archetype: Blood Pressure

The idea behind the design of an archetype-based Database schema was to incorporate the openEHR archetypes’ structure into a table schema with respect to HBase table-design logic. Thus, the mapping between the archetypes and an HBase table could be performed by following the next steps:

- **Definition of a table schema**, in which the column families are mapped to the labels contained into the archetype classes/subclasses described in the openEHR subsection and at the same time are used for the representation of concepts in a particular use-case scenario. If for example one should map only archetypes of the observation subclass to an HBase table, the column families would be “State”, “Events”, “Attribution”, “Data”, “Protocol”, and “Description” (see Fig. 1). However, the labels “Description” and “Attribution” should be excluded, as they contain common details related to the archetype and not to the concept they represent, such as original author, translators, purpose, use etc., and there is no point in storing such information for every record in the database. Hence, this information could be stored in a separate table.
- **Population of the column families with columns** for storage of the information represented by the archetype in a particular use-case scenario. Since usually the archetypes contain more fields than required for the representation of the corresponding concepts, columns have to be created only for the storage of data related to the used fields of the archetypes. Additionally, each archetype may require different table columns for its storage. If for example one would like to store the “Weight” and “Comment” fields of the label “Data” and “Any event” of the label “Events” for the representation of Body weight (Fig. 1), then three separate columns should be added to the HBase table as shown in Table 1. Similarly, if one would like to store additionally the “Systolic” and “Any event” fields for the representation of Blood pressure (Fig. 2), then more columns would be added to the HBase table.

**Table 1:** Example: Result of the archetypes - Hbase table schema mapping

Rowkey	Data				Events
	Systolic	Weight	Comment	Synopsis	Any Event
1001_20180312_EHR-OBSERVATION.blood_pressure.v1	143.0		Abnormal		High BP
1001_20180312_EHR-OBSERVATION.body_weight.v2		85.2	Normal		No
1001_20180422_EHR-EVALUATION.clinical_synopsis.v1				Synopsis text	
1001_20180613_EHR-OBSERVATION.body_weight.v2		92.3	Obesity		High weight gain
⋯
- **Determination of the rowkey structure** which will be common for all column families. The name of the archetype has to be included in the rowkey to avoid a possible overlap of data in cases where the fields of the used archetypes have common names. Thus, by including the archetype name into the rowkey, data related to different archetypes are stored in different rows. For example for the 4 sample entries of clinical data presented in Table 1, each row is identified by a unique rowkey which is composed by the patient identifier (eg. 1001), the date of the measurement in “YYYYMMDD” format (eg. 20180312), and the name of the archetype (eg. EHR-OBSERVATION.body_weight.v2) which describes the clinical entity. If we hadn’t included the archetype name into the rowkey, then the second row would have the same rowkey as the first one (1001_20180312), and thus the “Comment” as well as the “Any event” values of the first record would be overwritten.

## Results

### Entities included in the VPM

The requirement analysis of the project resulted in the definition of a VPM which includes information about frailty-related entities that cover every important aspect of an older person’ s health and life in the most meaningful way. In this way a coherent personalized profile is constituted and can be used for health status monitoring and age-related decline prevention. In Tables 2, 3, 4 and 5 all these entities can be seen in detail.

**Table 2:** Mapping between VPM personal details and Archetypes

Parameters	Parameters Content	openEHR Archetypes Existing/New	Archetype items Used/Added
Personal details	Patient Identification	EHR-CLUSTER.individual_personal.v1	Items.Identifier
	Demographic Details (Gender, Date of birth)	DEMOGRAPHIC-ITEM_TREE.person_details.v1	Data.(Birth date, Gender)
	Contact Details	DEMOGRAPHIC-ADDRESS.address.v1	Details.(Country identifier,...)
		EHR-CLUSTER.telecom_details.v0	Items.(Email Address,...)

**Table 3:** Mapping between VPM device-measured parameters (related to measurements from the FrailSafe devices) and Archetypes

Parameters	Parameters content	openEHR Archetypes Existing/**New**	Archetype items Used/**Added**
Heart rate	-Daily statistics (average,max, etc.) for: heart rate,rr-interval,heart rate variability when: 1)sitting/standing,2)walking,3)lying, 4)walking upstairs/downstairs, 5)in transition -Device used,-Abnormal Events	EHR-OBSERVATION.pulse.v1 EHR-CLUSTER.device.v1	Data.Rate (beats per min), **Data.Variability**, State.Position, Events.Maximum, Events.Any event, Protocol.device
		EHR-OBSERVATION.ecg_test_result.v0	Data.RR Rate
		EHR-CLUSTER.level_of_exertion.v1	Items.Exercise.Description (e.g. walking)
Respiration Rate	-Daily statistics (average,max, etc.) for: respiration rate, breathing amplitude when: 1)sitting/standing,2)walking,3)lying, 4)walking upstairs/downstairs, 5)in transition -Device used,-Abnormal Events	EHR-OBSERVATION.respiration.v1 EHR-CLUSTER.device.v1	Data.Rate (beats per min), Data.Depth **State.Position**, Events.Any event, **Protocol.device**
		EHR-CLUSTER.level_of_exertion.v1	Items.Exercise.Description (e.g. walking)
Blood pressure	-Mean daily systolic/diastolic/pulse value -Device used, -Abnormal Events	EHR-OBSERVATION.blood_pressure.v1 EHR-CLUSTER.device.v1	Data.(Systolic, Diastolic, Pulse Pressure)(mm[Hg]), Events.Any event, Protocol.device
Arterial stiffness	-PulseAmplification, Augmentation Index75, Vascular Resistance, Cardiac Output, Stroke Volume Cardiac Index, Augmentation, Reflection Coefficient, Pulse Wave Velocity, Mean stiffness value daily, -Device used	**EHR-OBSERVATION.arterial_stiffness.v0** EHR-CLUSTER.device.v1	Data.(Pulse Amplification,..., Stiffness), Protocol.Device
Indoor activities	Mean time spent daily: 1)in the living room, 2)in the restroom, 3)in the bedroom, 4)indoors in general, 5)walking inside, 6)sitting/standing, 7)lying	**EHR-OBSERVATION.activities.v0** EHR-CLUSTER.device.v1	Data.Duration (min), Data.Description, Data.Place, Protocol.Device
Outdoor mobility pattern	Daily values for: 1) total distance covered, 2) total duration, 3) total number of steps, 4) radius covered, 5) area covered, 6) average walk speed, 7) total walk time, 8) total stop time, 9) total vehicle time, 10) walk time percentage, 11) vehicle time percentage, 12) stop time percentage, 13) number of tracks, 14) track average distance, 15) track average duration, 16) track maximum distance, 17) track maximum duration	**EHR-OBSERVATION.outdoor_mobility_pattern.v0** EHR-CLUSTER.device.v1	Data.(Distance, Duration,...,Track maximum duration), Protocol.Device
Game1	Daily values for: 1) Max force, 2) Average max force, 3) Average endurance, 4) Max endurance, 5) Average score, 6) Max score, 7) Average game duration, 8) Max game duration Daily statistics for: 9) Height over game duration, 10) Distance over game duration, 11) Speed over game duration, 12) Lives over game duration, 13) Force over game duration	**EHR-CLUSTER.red_wings_game.v0**	Data.(Max Force,...,Force over game duration)

Bold text indicates newly developed archetypes or added archetype items

**Table 4:** Mapping between VPM clinical-measured parameters (related to measurements from clinical evaluations) and Archetypes

Parameters	Parameters Content	openEHR Archetypes Existing/**New**	Archetype items Used/**Added**
Nutrition	For each clinical assessment: Body Weight, Waist circumference, Body mass index, Body fat, Lean body mass, MNA [63] total score	EHR-OBSERVATION.body_weight.v2 EHR-OBSERVATION.waist_circumference.v1 EHR-OBSERVATION.body_mass_index.v1 EHR-OBSERVATION.body_composition.v1 EHR-OBSERVATION.body_mass_index.v1 **EHR-OBSERVATION.mna_questionnaire.v0**	Data.Body Weight (kg or lb) Data.Waist circumference (cm) Data.Body mass index Data.Fat mass Data.Body free mass index Data.Total score
Social interaction	1)Number of phone calls, 2)Number of text messages 3)Time spent speaking at the phone, 4)Time spent on video-conference 6)Living Conditions, 7)Number of leisure activities, 8)Membership in a leisure club	**EHR-OBSERVATION.** **generalities_questionnaire.v0**	Data.Phone Calls Data.Text Messages Data.Speaking Duration (min) Data.Video Conference Duration (min) Data.Living conditions, Data.Leisure activities, Data.Leisure club)
Cognitive State	MoCa [64], MMSE [65] questionnaires scores, subjective memory complain	**EHR-OBSERVATION.moca_questionnaire.v0** **EHR-OBSERVATION.mmse_questionnaire.v0** **EHR-OBSERVATION.** **cognitive_mood_sleep_questionnaire.v0**	Data.Total Score Data.Total score Data.Memory complain
Psychological State	GDS-15 [66] questionnaire score, self rated anxiety	**EHR-OBSERVATION.gds-15_questionnaire.v0** **EHR-OBSERVATION.** **visual_analogue_scale.v0**	Data.Total score Data.Score
Physical condition	Single foot standing), Time Get up and Go, Gait-speed (4m), Lower Limb strength, Low physical activity	**EHR-EVALUATION.** **gait_balance_evaluation.v0** **EHR-OBSERVATION.fried_criteria.v0**	Data.(Single foot standing, Time Get up and go, Gait speed 4m, Raise from the chair 5 times) Data.Low physical activity
Functional capacity	Score in Katz index [67] of ADL scale and score in Lawton’s ADL scale [68]	**EHR-OBSERVATION.katz_index_questionnaire.v0** **EHR-OBSERVATION.lawdon_adl_questionnaire.v0**	Data.Total Score Data.Total score
General condition	Unintentional weight loss, Self-reported Exhaustion	**EHR-OBSERVATION.fried_criteria.v0**	Data.Exhaustion, Weight loss
Wellness	Self-rated: Quality of life, pain, health status, change since last year	**EHR-OBSERVATION.** **visual_analogue_scale.v0**	Data.Score
		**EHR-OBSERVATION.health_self_rating.v0**	Data.(Health status, Change)
Lifestyle	Smoking, Alcohol Consuption, Physical activity	EHR-EVALUATION.tobacco_smoking_summary.v1 EHR-OBSERVATION.substance_use-alcohol.v1 EHR-OBSERVATION.physical_activity.v1	Data.Overall Status Data.(Frequency, Amount) Data.Physical activity level
Housing condition	Subjective suitability of the housing environment according to participant’s evaluation and investigator’s evaluation Number of steps to access the house	**EHR-OBSERVATION.housing_condition.v0**	Data.(Suitability (participant), Suitability (investigator), Number of steps)
Medical domain	Number of co-morbidities, Number of significant co-morbidities, Number of medications, Orthostatic hypotension, Vision, Hearing	EHR-EVALUATION.problem_diagnosis.v1 EHR-INSTRUCTION.medication_order.v2 EHR-COMPOSITION.report-result.v1	Data.(Problem/Diagnosis name, Severity) Activities.Order.Medication Context.Status
Frailty	Frailty phenotype categorization by Fried [62]	**EHR-OBSERVATION.fried_criteria.v0**	Data.Index

Bold text indicates newly developed archetypes or added archetype items

**Table 5:** Mapping between VPM events and interventions parameters, and Archetypes

Parameters	Parameters Content	openEHR Archetypes Existing/New	Archetype items Used/Added
Events	1) Events related to health issues 2)Notifications delivered to clinicians	EHR-CLUSTER-health-event.v0 EHR-INSTRUCTION.notification.v0	Items.Event Name, Items.Description Notification.Data.Category.T
Interventions	Health and lifestyle related interventions	EHR-EVALUATION.recommendation.v1	Data.Recommendation

More specifically, Table 2 consists of identification and demographic details which are collected once and remain unchanged or change rarely.

Table 3 consists of elements measured by the FrailSafe devices. FrailSafe devices and sensors record a large amount of data corresponding to several parameters like blood pressure, pulse waves velocity, heart and respiratory rate, physical strength and activity, postural and movement information, localization and cognitive performance.

Table 4 contains information about VPM entities that are monitored during clinical assessment sessions which take place once every six months. During these sessions the CGA [61] is employed, which is a laborious and generally time consuming complex procedure, initially analytic and eventually synthetic, requiring skills and expertise.

Table 5 includes i) events that are related to abnormal health measurements, and ii) recommendations that are given as input by the clinicians in cases of of abnormalities or unusual events. The events can trigger alerts responsible for the notification of the health care provider or the individuals’ family members.

### Description of the archetypes utilized

The adapted methodology resulted in the definition of 22 coherent and clinically meaningful parameters that cover every aspect of the concepts to be represented as shown in column “Parameters” in Tables 2, 3, 4 and 5. Furthermore, aiming at the comprehensive representation of the required concepts, each of the 22 parameters contains conceptually smaller objects used to express different aspects. For instance, the parameter “Heart Rate” which describes the general concept, consists of the following specialized objects: daily statistics (average,max, etc.) for heart rate, RR-interval, heart rate variability, position of the body or activity performed during the measurements; device used for the measurements, and any abnormal events occurred. These objects represent the project recordings about heart rate. Following the same process for each of the parameters, numerous specific objects were generated and are shown in the column “Parameters Content” in Table 3. As mentioned in the previous section, the next step of our methodology was to define a mapping between the aforementioned parameters and openEHR archetypes. This mapping resulted in:

1. Direct exploitation of 22 archetypes without any modification, as their fields captured comprehensively the concepts required for the complete representation of the corresponding parameters. As an example for this case, "EHR-OBSERVATION.blood_pressure.v1" archetype had all the necessary fields for the representation of the objects related to the parameter "Blood Pressure" (Fig. 2).
2. Modification and reuse of two archetypes. More specifically, the fields of the "EHR-OBSERVATION.
  respiration.v1" archetype represented almost all the required objects for the representation of the "Respiration Rate" parameter except for the position of the subject during the data acquisition and the device used for the measurement. So, this archetype had to be extended by adding a "State.Position" and "Protocol.Device" field using the openEHR archetype editor (Fig. 3).

**Fig. 3:**
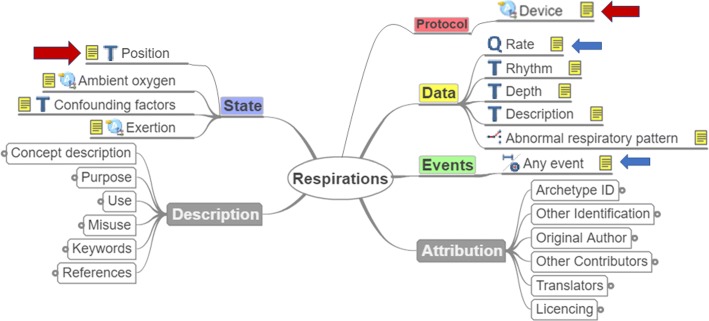
Example of modified archetype
3. Development of 28 new archetypes to represent the parameters related to frailty, that were not yet modeled by existing openEHR archetypes (Fig. 4). In Tables 2, 3, 4 and 5 only 19 of them can be shown as another 9 archetypes had to be designed for the representation of the rest of the games apart from Game1.

**Fig. 4:**
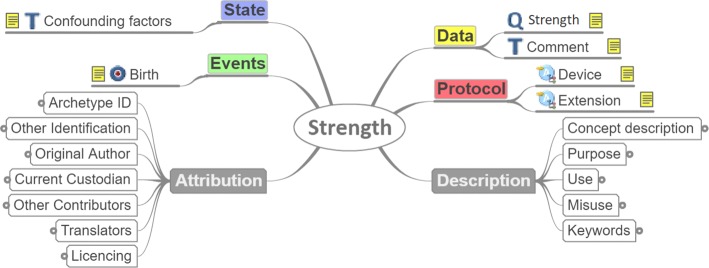
Example of developed archetype

### Produced NoSQL database

In this subsection, the NoSQL schema resulted from the methodology described in “Methods” section is discussed. As mentioned before, the column families of the table in which the VPM data are stored are mapped to the labels of the (reused, modified or developed) archetypes, as shown in Tables 2, 3, 4 and 5. For example, the required labels for the representation of "Blood Pressure" are: Data, State, Protocol, Events. These labels include all the required items of the incorporated archetypes, which in this case are: Systolic, Diastolic, Pulse, Position, Device, Any event. Following the same procedure for every parameter of Tables 2, 3, 4 and 5, the final column-family NoSQL schema of the produced Database is generated. Two different HBase tables are used for the storage of the data based on the nature of the stored information (subject-specific or temporal parameters), which consequently necessitates the use of corresponding tables for updates. More specifically:

- The first table is used for storing the personal details of the participants/patients, which are static and hence, rarely altered.
- The second table stores all the dynamically changing parameters, which are aggregated daily or at regular intervals.

## Conclusions

This work exploits the openEHR modeling approach with its reference model and archetypes for the representation of frailty in ageing population. A detailed personalized virtual patient model has been designed and developed, composed of older people’s clinical information and aggregated dynamic measurements acquired from embedded and wireless smart indoors and outdoors sensors. The use of this VPM, which can be considered as the older person’s virtual alter ego, aims at facilitating the development of reliable advanced intervention services and the determination of the risk of increasing frailty in older people. Furthermore, this paper provides a description of a methodology followed for a one-to-one mapping between openEHR archetypes (existing or newly developed) and a column-family NoSQL database. Finally, the aforementioned mapping methodology and the summary of archetypes (reused, newly developed and modified) from this work are shared in a public open source repository [52].

It is evident that our work is targeted to the frailty related entities. However, this should not be perceived as a limitation, as the same methodology can be easily adapted to new use cases. There are some fields, like the frailty status, which are specific to the older population, but most of the fields generalize for all age groups and several health conditions.

Our future work will focus on three main aspects. Firstly, the developed VPM and its implementation in HBase will be used as a base for the development of FrailSafe’s final Decision Support System (DSS). This system will be designed using the openEHR’s Guideline Definition Language (GDL) for expressing its logic, as interoperability is of main concern. Secondly, a visualization platform for the developed VPM and the corresponding DSS will be implemented. The implemented visual analytic techniques will deliver different perspectives on specific sub-graphs of the correlated data and models to provide role-specific (personalized) and goal-oriented representations of the medical information. Finally, the stored VPM data will serve as input in a series of machine learning methods that aim at extracting knowledge on frailty and its progression in ageing population.
